# Sero-prevalence of hepatitis B infection among blood donors in a secondary care hospital, Ghana (2014): a retrospective analysis

**DOI:** 10.1186/s13104-017-2733-3

**Published:** 2017-08-10

**Authors:** Eric Osei, Sylvester Yao Lokpo, Eric Agboli

**Affiliations:** 1 Department of Population and Behavioural Sciences, School of Public Health, University of Health and Allied Sciences, PMB 31, Ho, Volta Region Ghana; 2 Medical Laboratory Department, Municipal Hospital Laboratory Services, Ho Municipal Hospital, Ghana Health Services, Ho, Volta Region Ghana; 3 Department of Epidemiology and Biostatistics, School of Public Health, University of Health and Allied Sciences, Ho, Volta Region Ghana

## Abstract

**Background:**

The prevalence of transfusion associated hepatitis B virus infection varies across different geographical populations. Establishing the sero-prevalence of the disease is important to informing the direction of preventive and control strategies. We sought to estimate the sero-prevalence of hepatitis B surface antigen among blood donors in Ho Municipal Hospital, Ghana.

**Methods:**

This was a retrospective study which involved reviewing of blood donation records for the year 2014 in Ho Municipal Hospital. The records were analysed to determine the prevalence of hepatitis B virus among blood donors. Data analysis was done using STATA statistical package.

**Results:**

A total of 576 blood donors were screened in 2014, out of which 520 (90%) were males and the rest females. The overall sero-prevalence of hepatitis B virus was 7.5% (95% CI 5.6–9.9%). The prevalence was highest (8.9%; 95% CI 5.6–14.0) among donors between 30 and 39 years old and among females (14.3%; 95% CI 7.4–25.7). Females were about 2.5 times more likely to be HBsAg positive compared with males (p < 0.05).

**Conclusions:**

The findings suggest that the study region is of intermediate to high endemicity with hepatitis B infection. Generally, females are more likely to be HBsAg positive than males. Planning more extensive screening and vaccination campaigns and educational programmes would help reduce the transmission of the infection among the general population.

## Background

Hepatitis B infection is one of the commonest viral hepatitis throughout the globe and has infected about two billion people, including an estimated 350 million chronically infected persons [1]. The prevalence of hepatitis B infection varies geographically with endemic areas in sub-Saharan Africa and East Asia where about 5–10% of the adult population are affected [2]. The lifetime and mortality risks resulting from hepatitis B infection in these regions are estimated at about 60 and 25% respectively [2, 3].

Hepatitis B virus (HBV) is the major cause of viral hepatitis with associated clinical manifestations such as hepatomegaly, cirrhosis, and hepatocellular carcinoma [4]. An enveloped partial ssDNA virus infects the liver, causing hepatitis B infection [5, 6]. The virus replicates in the hepatocytes causing impairment in the functions of the liver [7]. The inflammation and the damage of the liver arise as a consequence of the immune response to the virus in the liver cells [8].

HBV may be present in whole blood, blood products and body fluids such as vaginal secretions, and in low concentrations in the saliva of active carriers [9]. The average incubation period of the virus is 90 days from the time of exposure to onset of symptoms, but may vary from 6 weeks to 6 months [10, 11].

In developing countries, unsafe blood is still a major issue probably due to the transmission of the virus through blood and blood products posing enormous public health concern [12]. In the West African sub-region, sero-prevalence levels ranging from 3 to 22% have been reported among blood donors [6, 13–15]. These results were similar to those of other studies in Asian countries with high endemicity [16, 17].

In Ghana, some studies have reported the prevalence of HBsAg among blood donors in health facilities; with estimated rates ranging from 10.8 and 11.6% in Northern Ghana, 11.8 and 6.9% in Ashanti Region to 15% in Greater Accra Region [18–20]. However, there is paucity of literature on the prevalence of HBsAg among blood donors in the Volta Region of Ghana. The current study therefore aimed at estimating the prevalence of hepatitis B infection among blood donors at the Ho Municipal Hospital in the Volta Region.

## Methods

### Study design and setting

This was a retrospective study conducted in Ho Municipal Hospital Laboratory among blood donors from January to December, 2014. Ho is the administrative capital of the people of the Volta Region. The municipality shares boundaries with the Republic of Togo to the east, to the west with Ho West District, to the North with Hohoe Municipality and to the south with Agortime–Ziope district. Ho has the largest population in the Volta Region with an estimated 214,612 inhabitants in 2014. Ho Municipal Hospital is one of the three state-owned hospitals located in the Ho Township [21]. Pentavalent HBV vaccination was incorporated into the early childhood vaccination in all regions in Ghana in the year 2002 and it is given in three doses at 6, 10 and 14 weeks of birth with coverage rate ranging from 77.9% in 2002 to over 90% in 2015 [22].

### Study population

The study included 567 replacement donors (individuals called upon to donate blood to patients) and voluntary donors (unpaid individuals who on their volition donated blood to the blood bank). Data source however did not segregate these two categories of donors. All blood donors were routinely screened for transfusion transmissible infections (TTIs) including HBV, HCV, HIV, and syphilis according to Ghana Blood Transfusion Services protocol before donations. The hepatitis B test strip used was based on immunoassay for determination of surface antigen for hepatitis B virus.

### Laboratory procedures

#### Blood collection

Venous blood was collected from the donors who presented at the blood bank unit of the laboratory. The blood was screened for anti-HIV, anti-syphilis, hepatitis b surface antigen (HBsAg) and anti-hepatitis C Virus.

#### Blood testing procedure

One Step hepatitis b surface antigen test strip (ACON Laboratories Inc. San Diego, CA) was used for screening donors’ serum samples. The test was based on the principle of immuno-chromatography. The procedure in obtaining test results was carried out according to the standard operating procedures which was based on manufacturer’s instruction in the package insert of the test strip. Briefly, donors’ whole blood sample was obtained, allowed to clot and centrifuged at 3000 revolutions per minute for 5 min at room temperature. The serum obtained was tested for the presence of surface antigen using the test strip. A positive test indicates viral infectivity while a negative result shows the absence of the virus. The test kit was CE (European Conformity) marked and FDA (Food and Drug Administration) approved.

### Data analysis

Data obtained was entered into Microsoft Excel spread sheet for cleaning and exported to Stata Version 11 (Stata Corp, Collage Station) statistical package for analysis. Descriptive analyses such as frequencies and cross tabulation of sex and other demographic variables were performed. Hepatitis B virus (HBV) status was analysed as a binary variable coded as ‘0’ for HBV positive and ‘1’ for HBV negative. The prevalence was estimated as the proportion of donors with HBV positive.

### Ethical consideration

Because hepatitis B screening is already routinely conducted as per the guidelines of the Ministry of Health for all blood donors, informed consent was not warranted. The data analysed had no identifiers for the donors and hence Scientific Receive Committee of School of Public Health, University of Health and Allied Sciences ruled that no formal ethics approval was required. Permission was however obtained from the hospital management to use the data for the purpose of research only.

## Results

A total of 576 blood donors were screened from January to December, 2014. Table 1 shows the distribution of blood donors by age and district of residence. The majority (289; 50.2%) were between 20 and 29 years, while adolescents (<20 years) constituted the least population of blood donors (16; 2.8%). More than 90% were males and 64% resided in the Ho municipality.

**Table 1:** Distribution of blood donors by age and district of residence stratified by sex, Ho Municipal Hospital, 2014

Variable	Male, n (%)	Female, n (%)	Total, N (%)
Age group (years)			
<20	11 (68.8)	5 (31.2)	16 (2.8)
20–29	265 (91.7)	24 (8.3)	289 (50.2)
30–39	160 (88.9)	20 (11.1)	180 (31.2)
≥40	84 (92.3)	7 (7.7)	91 (15.8)
District of residence			
Adaklu	46 (83.6)	9 (16.4)	55 (9.6)
Agortime-Ziope	10 (100)	0 (0.0)	10 (1.7)
Ho municipal	338 (91.6)	31 (8.4)	369 (64.1)
Ho west	96 (86.5)	15 (13.5)	111 (19.3)
Others	30 (96.8)	1 (3.2)	31 (5.4)
Total (N)	520 (90.3)	56 (9.7)	576 (100)

### Prevalence of hepatitis B

The overall prevalence of HBV among blood donors was 7.5% (95% CI 5.6–9.9%). The highest prevalence was recorded among the 30–39 years age group (8.9%), slightly higher than the 40 years and above age group (8.8%), and the least prevalence of 0% was observed among adolescent (<20 years) donors. Eight (14.3%) of the females were tested HBsAg positive as compared to 35 (6.7%) males. The prevalence was highest among females in all the age groups (Table 2). More than 58% (25/43) of all HBsAg positives donors were from the Ho municipality. However, the prevalence was highest among donors from Adaklu District (12.7%). The prevalence among donors from Agortime-Ziope, Ho Municipal, and Ho-West District were 10, 6.8, and 6.3% respectively (Table 3).

**Table 2:** Prevalence of hepatitis B infection among blood donors stratified by age group and sex

Age group (years)	Female, n/N (%)	Male, n/N (%)
<20	0/11 (0)	0/1 (0)
20–29	4/24 (16.7)	15/265 (5.7)
30–39	3/20 (15.0)	13/160 (8.1)
≥40	1/7 (14.3)	7/84 (8.3)

**Table 3:** Prevalence of hepatitis B among blood donors by demographic characteristics

Variable	# of donors	# of HBs Ag positives	Prevalence (%)	95% CI
Age group (years)				
<20	16	0	0.0	0.0–20.4
20–29	289	19	6.6	4.3–10.0
30–39	180	16	8.9	5.6–14.0
≥40	91	8	8.8	4.5–16.4
Sex				
Male	520	35	6.7	4.9–9.2
Female	56	8	14.3	7.4–25.7
District of residence				
Adaklu	55	7	12.7	6.3–24.0
Agortime-Ziope	10	1	10.0	1.8–40.4
Ho municipal	369	25	6.8	4.6–9.8
Ho west	111	7	6.3	3.1–12.5
Others	31	3	9.7	3.4–24.9
Total	576	43	7.5	5.6–9.9

## Discussion

Blood transfusion is an important component of health care and globally, millions of lives are being saved each year through this procedure [23]. In Ghana, scores of people visit the blood banks of various hospitals and other blood donation centres across the country to donate blood to their dear ones who need blood for survival. This study showed that most donors were males and were aged between 20 and 38 years. This observation is similar to earlier reports from the Ashanti and Northern Regions of Ghana, and in Western Uttar Pradesh, India [18, 19, 23, 24]. Ampofo et al. [20] observed that the trends of male bias in blood donation is a regular feature commonly experienced among Ghanaians during blood donation campaigns. Though the reason for this phenomenon may not be inferred directly from this study, it is thought that certain socio-cultural influences and beliefs may play a major role [24]. Dongdem et al. [18] posited that within the Ghanaian context, men are more likely to take action based on an independent decision to save a life by volunteering to donate blood than their female counterpart.

The prevalence of hepatitis B infection among blood donors observed in this study can be described as that of intermediate to high endemicity [25] and is similar to what Nkrumah et al. [19] found in their previous study at Agogo Presbyterian Hospital in the Ashanti Region of Ghana. Our observed prevalence is however relatively lower than what were previously reported in some parts of Ghana such as, Accra (15%) [20], Tamale (10.8–11.6%) [18], and Kintampo (9.6%) [24]. It is important to note that the aforementioned studies were done years before our study and since those studies, a remarkable achievement have been made by Ghana AIDS commission through efforts such as increasing access to counselling and testing services for sexually transmitted diseases, routine screening of antenatal attendants for HIV, Syphilis and hepatitis B infections through the Prevention of Mother-to-Child Transmission programme, and the expansion of school based HIV education campaign, to reduce the incidence of sexually transmitted diseases in general reflecting the reduction of the median HIV prevalence among the general Ghanaian population from 3.6% in 2003 to 2.1% in 2012 [26]. This could explain the lower prevalence of hepatitis B infection among blood donors observed in our current study compared with previous report in some parts of the country.

The highest prevalence was observed among donors over 30 years old. There was no donor less than 20 years old who tested HBsAg positive. This finding is inconsistent with earlier reports from other parts of Ghana such as Kintampo [24] and Tamale [18] where the prevalence was highest among younger donors. Low HBsAg detected among younger donors in this study might most probably be due to the fact that transmission of HBV at the site is through heterosexual contact rather than vertical transmission from mother-to-child. Our finding is however in consonance with other studies from varied populations [27, 28]. This could suggest that this category of individuals may not have been vaccinated against hepatitis B, a situation observed to be common among developing countries [18].

Available literature on few studies in Ghana with regards to hepatitis B infection among donor populations stratified by sex have reported inconsistent results. Nkrumah et al. [19] observed in their study that hepatitis B infection was more prevalent among female donor population than males, whereas Walana et al. [24] and Dongdem et al. [18] observed a greater donor male population with infected blood. In our current study, the prevalence of HBV infection was estimated to be highest among females in all the age groups. There may be no apparent specific reason attributed to this difference but other studies also reported similar differences among sex. In Namibia, males were more positive for Hepatitis B than females [29].

The prevalence of HBV infection varies from among different geographical districts in and around the study site from where the blood donors resided. Even though relatively the blood donor population was fewer among rural districts (Adaklu and Agortime-Ziope), the prevalence of HBV was found to be highest among donors from these districts. The might be due to increased exposure to risk factors in conjunction with poor sanitary and socioeconomic condition in the rural areas. These findings may however not precisely reflect the prevalence of the blood donor population due to donor selection processes involved.

## Conclusions

We can conclude that this study has provided information on the burden of HBV infection in Ho, Ghana. The findings suggest that, though the HBV prevalence among blood donors is relatively low compared with previous reports from other parts of Ghana, it still remains within high endemicity, with the highest prevalence recorded among females. More extensive screening and vaccination campaigns within the general adult population as well as educational programmes on the risks and benefits of immunization should be embarked upon in an effort to reduce the transmission rate of the infection. Better reflection of the sero-prevalence of HBV infection in this region of Ghana may be studied in the general population.

## Abbreviations

AIDS: acquired immune deficiency syndrome
HBsAg: hepatitis b surface antigen
HBV: hepatitis b virus
HIV: human immunodeficiency virus
